# Flumequine (Veterinary Medicinal Products)

**DOI:** 10.14252/foodsafetyfscj.D-20-00005

**Published:** 2020-03-27

**Authors:** Food Safety Commission of Japan

## Abstract

FSCJ conducted a risk assessment of an antimicrobial, flumequine (CAS No. 42835-25-6), based on reports of JECFA (Joint FAO/WHO Expert Committee on Food Additives) and EMEA (European Medicines Agency) and other documents including the mechanism for liver tumor. Data used in the assessment include pharmacokinetics, acute toxicity, subacute toxicity, chronic toxicity/carcinogenicity, reproductive toxicity, genotoxicity, and microbiological effects. FSCJ specified the ADI of flumenquine as 0.071 mg/kg bw per day, that is the microbiological ADI calculated using the equation for VICH.

## Conclusion in Brief

FSCJ conducted a risk assessment of an antimicrobial, flumequine (CAS No. 42835-25-6), based on reports of JECFA (Joint FAO/WHO Expert Committee on Food Additives) and EMEA (European Medicines Agency) and other documents including mechanism for liver tumor.

Data used in the assessment include pharmacokinetics (rats and dogs), fate and residues (cattle, sheep, pigs, chickens, rainbow trouts and shrimps), residues (turkeys), acute toxicity (mice, rats, rabbits and dogs), subacute toxicity (mice, rats, guinea pigs, dogs and monkeys), chronic toxicity/carcinogenicity (mice, rats and dogs), reproductive toxicity (mice, rats and rabbits), genotoxicity, and microbiological effects.

In an *in vivo* comet assay, a positive result on genotoxicity was obtained in liver. Negative results were, however, obtained in the liver of two *in vivo* gene mutation studies using *gpt* delta mice. These results suggested the damaged DNA was repaired prior to induction of mutation. Therefore, FSCJ concluded that flumequine has no genotoxicity relevant to human health.

Although tumor development in the liver was observed in mice, FSCJ presumed that genotoxicity mechanisms were not involved in this tumor development since flumequine had no genotoxicity relevant to human health. In one study in *gpt* delta mice, flumequine administration did not increase the concentration of 8-hydroxyguanosine but increased numbers of 5-bromo-2’-deoxyuridine positive cells. In another study in *gpt* delta mice, the increased levels of mRNA for cell cycle-related genes and also for cytokine genes were observed.

On the basis of these findings, the chronic liver injury, followed by activated cell proliferations, is rather associated with the flumequine-induced mechanism for liver-tumor development. FSCJ thus judged that an ADI is able to be specified.

Decreased body weights of fetuses and offspring were the major reproductive-developmental toxicities of flumenquine. Teratogenicity was not observed.

Toxicological ADI was specified as 0.25 mg/kg bw per day by applying a safety factor of 100 to the NOAEL of 25 mg/kg bw per day, which was obtained in 13-week subacute toxicity studies in mice.

Microbiological ADI was calculated to be 0.071 mg/kg bw per day using the equation for VICH.

FSCJ specified the ADI of flumenquine as 0.071 mg/kg bw per day as the microbiological ADI is smaller than the toxicological ADI.

